# SPECT-CT Imaging of Dog Spontaneous Diffuse Large B-Cell Lymphoma Targeting CD22 for the Implementation of a Relevant Preclinical Model for Human

**DOI:** 10.3389/fonc.2020.00020

**Published:** 2020-02-07

**Authors:** Floriane Etienne, Maxime Berthaud, Frédérique Nguyen, Karine Bernardeau, Catherine Maurel, Caroline Bodet-Milin, Maya Diab, Jérôme Abadie, Valérie Gouilleux-Gruart, Aurélien Vidal, Mickaël Bourgeois, Nicolas Chouin, Catherine Ibisch, François Davodeau

**Affiliations:** ^1^CRCINA, INSERM, CNRS, Université de Nantes, Université d'Angers, Nantes, France; ^2^AMaROC, Oniris (Nantes Atlantic College of Veterinary Medicine, Food Science and Engineering), Nantes, France; ^3^P2R “Production de Protéines Recombinantes”, CRCINA, SFR-Santé, INSERM, CNRS, UNIV Nantes, CHU Nantes, Nantes, France; ^4^Nuclear Medicine, University Hospital, Nantes, France; ^5^EA7501, GICC, Université de Tours, CHRU de Tours, Tours, France; ^6^Groupement d'Intérêt Public ARRONAX Cyclotron, Saint-Herblain, France

**Keywords:** comparative oncology, dog, diffuse large B-cell lymphoma, monoclonal antibody, SPECT-CT imaging, CD22, internalization

## Abstract

Antibodies directed against CD22 have been used in radioimmunotherapy (RIT) clinical trials to treat patients with diffuse large B-cell lymphoma (DLBCL) with promising results. However, relevant preclinical models are needed to facilitate the evaluation and optimization of new protocols. Spontaneous DLBCL in dogs is a tumor model that may help accelerate the development of new methodologies and therapeutic strategies for RIT targeting CD22. Seven murine monoclonal antibodies specific for canine CD22 were produced by the hybridoma method and characterized. The antibodies' affinity and epitopic maps, their internalization capability and usefulness for diagnosis in immunohistochemistry were determined. Biodistribution and PET imaging on a mouse xenogeneic model of dog DLBCL was used to choose the most promising antibody for our purposes. PET-CT results confirmed biodistribution study observations and allowed tumor localization. The selected antibody, 10C6, was successfully used on a dog with spontaneous DLBCL for SPECT-CT imaging in the context of disease staging, validating its efficacy for diagnosis and the feasibility of future RIT assays. This first attempt at phenotypic imaging on dogs paves the way to implementing quantitative imaging methodologies that would be transposable to humans in a theranostic approach. Taking into account the feedback of existing human radioimmunotherapy clinical trials targeting CD22, animal trials are planned to investigate protocol improvements that are difficult to consider in humans due to ethical concerns.

## Introduction

Radioimmunotherapy (RIT) using an anti-CD20 antibody radiolabeled with yttrium-90 (ibritumomab tiuxetan/Zevalin®) is approved for the treatment of patients with relapsed or refractory follicular lymphoma (FL) or in consolidation after a front-line induction chemotherapy (1–4). Other clinical trials in the field of B-cell lymphoma RIT aim at demonstrating the value of RIT in front-line treatment (5, 6), high-dose treatment (7, 8), and fractionated RIT protocol with humanized monoclonal antibody (MAb) (9, 10) or using new monoclonal antibodies (MAbs) specific for lymphoma antigens (11, 12). Recently, antibodies directed against CD22 have been used in RIT clinical trials to treat patients with diffuse large B-cell lymphoma (DLBCL) and B-cell acute lymphoblastic leukemia (B-ALL) with encouraging results (13, 14). But performing RIT protocols in first-line treatment is not currently feasible until further clinical data is obtained regarding safety and efficacy in later-line treatment. For these reasons, relevant preclinical models are needed to facilitate the evaluation and optimization of new protocols. The relevance of rodent models of lymphoma is limited to the small number of lymphoma cell lines that are able to grow in vivo. Indeed, these few cell lines do not represent the large physiopathological diversity of human tumor subtypes and the inter-individual heterogeneity of patients (15, 16).

To improve the relevance of the preclinical approaches, pet dogs with spontaneous lymphomas diagnosed in veterinary practice would be an asset. Most of the B-cell lymphomas diagnosed in dogs are DLBCLs (17). Among NHLs, DLBCLs are more aggressive than FL and new therapeutic approaches are needed for relapsing patients. In humans, phase I/II clinical trials targeting CD22 for DLBCL therapy are promising. Spontaneous DLBCL in dogs is therefore a tumor model that may help to accelerate the development of new methodologies and therapeutic strategies with enhanced probability of success when transferred to the clinic (18, 19).

Whole-body molecular imaging of dogs with SPECT (single-photon emission computed tomography) or PET (positron emission tomography) using radiolabeled antibody specific for tumor antigens is non-invasive and enables to more accurately evaluate the disease extension at diagnosis before setting conventional treatment of dogs with DLBCL. In a theranostic approach, molecular quantitative imaging enables the calculation of the actual dose deposition to organs within the course of RIT performed with the same anti-CD22 antibody. This personalization of treatment requires defining specific methods such as population pharmacokinetics to evaluate the individual pharmacokinetic profiles of the patients treated (20, 21). Setting up these approaches requires sequential imaging, which is difficult to impose on human patients, but is realistic in the veterinary clinic. Since spontaneous tumor imaging in humans and dogs is performed using the same camera, the transfer from veterinary to human clinical trials will be facilitated. Based on the most promising phase I/II clinical trial of DLBCL RIT targeting CD22 in humans (14), it is relevant to perform clinical trials on sick dogs focusing on the rationale of dosing or treatment schedule with the hope of therapeutic benefit compared to conventional chemotherapy. Chemotherapy applied to dogs is adapted from human treatments. A current treatment of lymphoma in dogs includes L-asparaginase, vincristine, cyclophosphamide, prednisone, and doxorubicin (COPLA) induction followed by chlorambucil, vincristine, and prednisone (LVP). This treatment, however, rarely cures dogs and the median survival after chemotherapy is only 6 months. Finally, the less limiting ethical constraint in veterinary medicine and the possibility of proposing clinical trials for dogs whose owner opts for corticosteroid therapy may facilitate the evaluation of RIT in front-line treatment, while offering the animals and owners the opportunity to benefit from cancer treatment unavailable in the veterinary clinic, but currently in validation for human patients.

To develop the model of spontaneous DLBCL in dogs for molecular imaging and RIT, we isolated seven mouse monoclonal antibodies directed against the canine CD22 (CD22c) antigen. Here we describe the characterization and selection strategy of these antibodies for future molecular imaging and RIT in dogs. A dog with spontaneous DLBCL was subjected to a SPECT-CT imaging with an indium-111-radiolabeled anti-CD22 antibody as part of disease staging in order to obtain the proof of concept of the relevance of this antibody in a veterinary clinical trial.

## Materials and Methods

### Cell Lines

The Chinese Hamster Ovary dihydrofolate reductase-deficient cell line (CHO DHFR^−^) purchased from ECACC (European Collection of Cell Cultures, ref 94060607) was transfected for stable expression of soluble membranous canine CD22 (CD22c). CHO wild type (WT) and DHFR- cells were cultured in Roswell Park Memorial Institute medium 1640 (RPMI 1640) (Gibco BRL) containing 10% fetal bovine serum (Gibco BRL, ref 10270106), 1% glutamine (L-glutamine 200 mM; Gibco BRL, ref 25030149), and 1% antibiotic (penicillin 100 U/mL, streptomycin 100 U/mL; Gibco BRL, ref 15140148), and supplemented with 10 μg/mL of adenosine-deoxyadenosine-thymidine (ADT) (Sigma-Aldrich, ref T-1895). The CLBL-1 cell line is a canine diffuse large B-cell lymphoma cell line kindly supplied by Rütgen et al. (22). All cell lines were incubated at 37°C in a humidified atmosphere in 5% CO_2_.

### Antibodies

The 6H4 MAb, a mouse IgG_1_ specific for human beta-2-microglobulin (β2m) produced and characterized in the laboratory, was used to screen and purify the canine CD22-human β2m fusion protein (CD22-β2m) as previously described (23).

A mouse anti-GFP monoclonal antibody [GFP Antibody (B-2); Santa Cruz Biotechnology, ref sc-9996] was used for Western blots using cell lysates of CD22c-GFP transfected CHO clones to detect the expression of the fusion protein CD22-β2m.

### Vectors and Genes

The pKCR6 vector was used as an expression vector for CHO cell transfection (24). The soluble CD22c fused to human β2m as well as the entire canine CD22c-GFP coding sequences were synthesized by GeneCust (Dudelange, Luxembourg). These sequences were received in a pBluescript II SK^+^ vector with a *Xho I* and *Xba I* enzyme restriction sites at their 5′ end and 3′ end, respectively.

### Production and Purification of CD22c-β2m Recombinant Protein

The soluble CD22c was produced as a fusion protein consisting of the ectodomain of CD22c (amino acids 1–683) fused to the human Beta-2-microglobulin (β2m) without the peptide signal (amino acids 21–119) via a linker of 15 amino acids constituted of three repeated (SerGlyGlyGlyGly)_3_ motifs. The coding sequence of the soluble form of CD22c merged to β2m (CD22c-β2m) was cloned into the pKCR6 vector and transfected into CHO cells using Lipofectamine™ LTX Reagent with PLUS™ Reagent (Invitrogen, ref 15338030) according to the supplier's instructions. Cells were then cultured in 96-well plates in ADT-free RMPI medium to select transfected cells. The supernatant of the wells with surviving cells 2–3 weeks post-transfection were tested for the expression of CD22c-β2m with an ELISA assay. Briefly, the supernatants of growing clones were diluted in PBS and coated on Nunc MaxiSorp™ plates (ThermoFisher Scientific, ref 44-2404-21). Non-specific sites were blocked with 100 μL of PBS-0.5% BSA. The biotinylated anti-β2m antibody 6H4 was added to each well followed by 50 μL of horseradish peroxidase (HRP)-conjugated streptavidin (R&D Systems, ref DY998) and tetramethylbenzidine (TMB) substrate (R&D Systems, ref DY999). The reaction was stopped with 1 M of sulfuric acid (H_2_SO_4_). Optical density (OD) was measured at 405 and 570 nm by a spectrophotometer (Multiskan EX, Thermo Scientific, Ventana, Finland). The cells from the positive wells were then subcloned by limiting dilution. The supernatant of the clones was screened with the same ELISA assay, in order to select high-expressing CD22c-β2m clones.

The transfected CHO cell clone showing the highest production of canine CD22-β2m fusion protein was selected and expanded to produce 1 L of culture supernatant. The recombinant protein was then purified by affinity chromatography using HiTrap NHS-activated HP affinity column (GE Healthcare, ref 17071701) coated with the 6H4 antibody according to the supplier's instructions. Eluted canine CD22-β2m protein was further purified by size-exclusion chromatography (Superdex 200 10:300GL, GE Healthcare) and the fractions containing high CD22-β2m were pooled, concentrated using an ultrafiltration Amicon Ultra-15 membrane (30K—Millipore). Purified canine CD22-β2m fusion protein was then sterilized by filtration over a 0.22-μm Minisart® Syringe Filter (Sartorius, ref 16534) and stored in PBS at −20°C.

### Gel Electrophoresis and Western Blotting

The purity of the CD22c-β2m protein produced was monitored by sodium dodecyl sulfate polyacrylamide gel electrophoresis (SDS-PAGE) with 5 μg of purified CD22c-β2m and human β2m (Sigma-Aldrich, ref M4890) used as a control. After electrophoresis on a 12% acrylamide gel under non-reducing conditions in 1x Tris Glycine SDS running buffer, the proteins were stained using Coomassie brilliant blue (National Diagnostics, ref HS-604) and the gel was scanned using a Bio-Rad scanner.

The SDS-PAGE gel was then transferred onto pore polyvinylidene fluoride (PVDF) membrane (Roche, ref 03010040001) in tris-glycine blotting buffer (Bio-Rad, ref 1610771). Non-specific sites were blocked using a TBS-0.1%-Tween-5% milk buffer, and PVDF membrane was incubated with the primary antibody 6H4 directed against human β2m for 2 h at room temperature. After washing, the membrane was incubated with a peroxidase-conjugated goat anti-mouse secondary antibody (Jackson ImmunoResearch, ref 115-035-003) (1:1,000 dilution) for 90 min. Target proteins were visualized with the Bio-Rad camera after revelation with the 3,3′-Diaminobenzidine (DAB) substrate.

### Production of CHO Cells Expressing Membranous Canine CD22 Fused to GFP

The full-length CD22c coding sequence (aa 1–848) with a *Xho I* restriction site at the 5′ end and a *Bgl II* restriction site at the 3′ end was produced by GeneCust laboratories (Luxembourg) and cloned into the pCR™ 2.1-TOPO^R^ plasmid using the TOPO™ TA Cloning™ Kit (Invitrogen, ref K456001). The *Xbo I-Xba I* CD22c coding sequence was then inserted in pEGFP-N3 (Clontech) and digested by *Xho I* and *Bgl II* in order to merge the CD22c and EGFP coding sequences. The CD22c-EGFP coding sequence in pEGFP-N3 and the expression vector pKCR6 were then digested with *Xho I* and *Xba I*. The fragments of interest were purified and ligated. The pKCR6/CD22c-EGFP construct was then transfected into Chinese hamster ovary (CHO) cells using the Lipofectamine™ LTX Reagent with PLUS™ Reagent (Invitrogen, ref 15338030).

The transfected cell line was subcloned by limiting dilution and the clones with positive green fluorescence were selected by flow cytometry analysis. Cell lysates from the highest expressing clones were further analyzed with Western blot using an anti-EGFP antibody (B-2). The clones with a positive band at the expected size corresponding to CD22c-EGFP were amplified and used thereafter for hybridoma supernatant screening.

### Mice Immunization Procedure

Three BALB/c JRj mice obtained from JANVIER laboratories (France) were immunized with the purified canine CD22-β2m recombinant protein. For each mouse, two immunizations 3 weeks apart were administered intraperitoneally with a constant amount of 50 μg of CD22c-β2m protein in PBS. These injections were performed with an equivalent volume of Freund's complete adjuvant (first injection) or incomplete adjuvant (second injection) (Sigma-Aldrich, ref F5881 and F5506) according to the supplier's instructions. One week after the second injection, blood was collected and antibody titers were assessed by flow cytometry analysis on the selected CD22c-EGFP expressing the CHO clone. The best responding mouse was selected and received a last intravenous boost of 50 μg of canine CD22c-β2m protein in PBS without adjuvant. Five days later, the mouse was sacrificed by cervical dislocation, the spleen was collected and splenocytes were harvested in RPMI 1640 medium for fusion. All experiments were conducted according to the National Institutes of Health (NIH) guidelines for handling experimental animals.

### Hybridoma Cell Production

Hybridomas were generated by the fusion of spleen cells from the immunized mouse with murine myeloma cells SP2/0 (ATC, ref PTA-5817) at a 5/1 ratio using 50% polyethylene glycol (PEG 1500) (Roche, ref 10783641001) according to the supplier's instructions. Hybridomas were grown in RMPI culture medium containing 20% fetal bovine serum, penicillin (100 U/mL) and streptomycin (100 U/mL) and supplemented with interleukin-6 (50 U/mL) and 2% hypoxanthine-aminopterin-thymidine (HAT) (ATCC) for the selection of hybridomas. This medium was replaced after 1 week with 2% hypoxanthine-thymidine supplemented medium (ATCC). Ten to 15 days after fusion, hybridomas were established in the wells and culture supernatants were screened.

### Hybridoma Supernatant Screening

The production of CD22c-specific antibodies was determined by flow cytometry analysis using the selected CHO cell clone expressing canine CD22c-EGFP. Hybridoma supernatants diluted in PBS-BSA 0.1% (1:1) were incubated with the canine CD22c-EGFP-positive CHO clone for 1 h at 4°C. A phycoerythrin-conjugated anti-mouse IgG (Jackson ImmunoResearch, ref 115-116-071) was used as a secondary antibody. Data acquisition and analysis were performed in a Becton Dickinson FACSCalibur flow cytometer (BD Biosciences) using FlowJo software (FlowJo LLC). The specificity of hybridomas for canine CD22c was confirmed using a similar flow cytometry method on WT CHO cells used as a negative control.

### Monoclonal Antibody (MAb) Purification and Isotype Determination

Selected cloned hybridomas were cultured in RPMI 1640 medium supplemented with 10% IgG-depleted fetal bovine serum in a 1-L Erlenmeyer flask (Corning®) with stirring at 80 rpm for 4 days at 37°C and 5% CO_2_ in an agitator. Clone supernatant cultures were collected and canine CD22-specific antibodies were purified over a HiTrap Protein G HP column (GE Healthcare, ref 29-0485-81). Briefly, supernatants from hybridoma cultures were diluted in phosphate buffer (1:1) to adjust the pH to 7. After passage through the column, antibodies were eluted using a glycine-HCl buffer pH 2.7 and dialyzed overnight against PBS pH 7.4 using 30,000 MWCO dialysis cassettes (Thermo Scientific). Purified antibodies were filtered through 0.2-μm filters and stored at 4°C and their production yields were determined.

Isotypes and light chains of purified antibodies were characterized using the IsoStrip™ Mouse Monoclonal Antibody Isotyping Kit (Roche, ref 11493027001) according to the kit instructions.

### Determination of MAb Equilibrium Dissociation Constant (Kd)

The affinity of MAbs was determined by flow cytometry as described above. For each antibody, a series of concentrations from 4.10^−7^ to 3.10^−11^ M was tested with a constant number of 2 × 10^5^ of the canine CD22c-EGFP-positive CHO cells. IgG1/K and IgG2b/K antibodies were used as control isotypes. Mean fluorescence intensities (MFI) were plotted against their corresponding antibody concentrations. Antibody dissociation constants were determined by a non-linear regression using the Prism software package (GraphPad Software Inc.).

### Epitope Mapping

Competition tests using the indirect ELISA method were performed to evaluate the number of distinct epitopes recognized by the antibodies produced. For these tests, 1 mg of each antibody was biotinylated using an EZ-Link™ Sulfo-NHS-Biotinylation Kit (Thermo Scientific, ref 21425) according to the supplier's instructions. Antibody biotinylation was essential for revelation with Streptavidin-HRP. An ELISA plate was coated with 250 ng/well of the anti-β2m antibody (6H4). Non-specific sites were blocked with PBS-0.5% BSA and 125 ng/well of the CD22c-β2m was added. Each biotinylated antibody was separately incubated at a constant concentration, equal to its Kd, with a 100-fold excess of each of the unlabeled antibodies. Then the wells were washed three times and revelation was finally performed with 50 μL of horseradish peroxidase (HRP)-conjugated streptavidin, as described above.

For the analysis of the competition between antibodies, the wells containing biotinylated antibody with an excess of a mouse IgG control isotype were considered as controls of the absence of competition. Wells containing the same antibody in biotinylated and unlabeled forms were considered as positive controls of the competition.

### Internalization

To estimate the internalization of anti-CD22 antibody, the CD22 proteins at the cell surface were quantified using the CLBL-1 cell line. We seeded 1.5 × 10^5^ CLBL-1 cells in two 96-well plates. One plate was kept at 4°C and the other at 37°C. At 0, 15, 30, 60, 90, 120, 180, and 240 min after incubation, each anti-CD22 antibody produced was added to the wells of the plate at 4 and 37°C. Multiple assays were performed with antibody concentrations corresponding to 10-, 5-, 1-, and 0.1-fold their own Kd. At the end of the incubation time, the plates were placed on ice, the cells were washed twice with ice-cold PBS and stained with phycoerythrin goat anti-mouse F(ab)′2 at 4°C for 1 h. After washing in ice-cold PBS BSA 0.1%, cell fluorescence was measured using a FACSCalibur cytometer. For each incubation time, the percentage of internalization was calculated as = [(MFI at 4°C)–(MFI at 37°C)]^*^100/(MFI at 4°C).

To evaluate the percentage of internalization as a function of the antigenic site saturation, the maximum binding of antibodies (Bmax) was evaluated for each antibody at each concentration after 240 min at 4°C at a saturating amount of antibodies using non-linear regression (one phase exponential association equation, PRISM GraphPad software). The actual percentage of saturated CD22c antigenic sites was also calculated for each antibody concentration 240 min after incubation at 4°C. The corresponding percentage of internalization was then plotted against the percentage of saturation of cell-surface CD22c.

### Immunohistochemistry

Formalin-fixed, paraffin-embedded (FFPE) 5-μm-thick tissue blocks from dogs were cut for anti-CD22 immunohistochemistry (IHC). A normal dog lymph node was used to check immunoreactivity of the canine CD22-specific clones produced. Samples of canine diffuse large B-cell lymphomas consisted of 30 previously diagnosed cases, of which 10 were of the germinal-center phenotype (i.e., positive for CD10, or CD10-negative but positive for BCL6 expression and negative for MUM1), and 20 were of the non-germinal-center phenotype (i.e., negative for CD10 and BCL6, or CD10-negative but positive for both BCL6 and MUM1). All histologic slides were freshly cut before IHC analysis. Briefly, sections were dried at 60°C for 2 h, deparaffinized, pretreated at 95°C for 60 min in a basic buffer (CC1, cell conditioning medium-1, pH 8.4; Ventana Medical Systems, ref 950-124) for antigen retrieval, and stained at 37°C for 60 min. The antibody concentrations were 2 μg/mL for the CD22-specific clone 2D1, 10 μg/mL for the clones 1E3, 5C2, and 5F8, 15 μg/mL for the clones 5A3 and 10C6, and 20 μg/mL for the clone 6B7. All dilutions were performed in a commercially available antibody diluent (Ventana Medical Systems, ref 251-018). All IHC protocols were run in a Ventana BenchMark XT immunostainer (Ventana Medical Systems). Antibody incubation was followed by chromogenic detection with the OptiView DAB IHC detection kit (Ventana Medical Systems, ref 760-700), counterstaining with 1 drop of hematoxylin-II for 4 min and post-counterstaining with 1 drop of Bluing Reagent for 4 min. Subsequently, slides were removed from the immunostainer, washed in water with a drop of dishwashing detergent, and mounted. In each run, a negative control was obtained by replacing the primary antibody with normal mouse serum (Negative Control Monoclonal Ig, 1 μg/mL, Ventana Medical Systems).

### Radiolabelling of Anti-CD22c MAbs

#### Radiolabeling of Anti-CD22c MAbs With Iodine-125

Anti-CD22c MAbs were labeled with ^125^I (Perkin Elmer, ref NEZ033001) using the iodogen method, as previously described (25). The ^125^I-labeled anti-CD22c MAbs were purified on a PD10 desalting column with sephadex G-25 (GE Healthcare, ref 17085101). Radiolabeling efficiencies, estimated by Instant Thin Layer Chromatography (ITLC), were above 95%.

#### Radiolabeling 10C6 MAb With Copper-64 and Indium-111

The 10C6 anti-CD22c clone was modified with p-SCN-Bn-DOTA (p-SCN-Bn-DOTA; Macrocyclics, ref B-205), as previously described (26). In brief, 10C6 MAb was incubated with 20 equivalents (mole/mole) of p-SCN-Bn-DOTA in borate buffer (0.2 M, pH 8.7) for 1 h at room temperature. The excess p-SCN-Bn-DOTA was removed by several filtration cycles on a centrifugal filter (Ultracel 30K, Amicon) using sodium acetate (0.2 M, pH 6). 10C6-DOTA was then radiolabeled with ^64^Cu (ARRONAX cyclotron) by adding 50 MBq ^64^Cu and adjusting the pH to 5.5 with sodium acetate (0.5 M, pH 5). The resulting ^64^Cu-labeled 10C6-DOTA was separated from unbound ^64^Cu by size-exclusion chromatography using a PD-10 column. Radiochemical purity, checked by ITLC, was >95%. The specific activity after purification was 216 MBq.mg^−1^.

The 10C6 Mab was radiolabeled with indium-111 according to the same protocol as that used for radiolabeling with ^64^Cu. Briefly, 1.0 mg 10C6-DOTA was mixed with indium-111 chloride adjusted to pH 5.5 with sodium acetate (0.5 M, pH 5) and incubated. The resulting ^111^In-labeled 10C6-DOTA was separated from unbound ^111^In by size-exclusion chromatography using a PD-10 column. Radiochemical purity, checked by ITLC, was 81%. The specific activity after purification was 128.8 MBq.mg^−1^.

### Xenogeneic Mouse Model of Canine DLBCL

All experiments were conducted according to the National Institutes of Health (NIH) guidelines for handling experimental animals (ethics committee of Pays de la Loire, CEEA 00143.01 and CEEA 2012.171).

#### Mouse Tumor Model

5.10^6^ CLBL-1 cells (canine B-cell lymphoma cell line) in 0.1 mL PBS were injected in the flank of 8-week-old NMRI-nu female mice (JANVIER). Biodistribution and PET imaging were carried out 14 days after tumor cell injection.

#### Biodistribution of ^125^I-Anti-CD22c MAbs

Biodistribution was carried out by injecting mice in the tail vein with 6 μg of ^125^I-anti-CD22c MAbs in 0.1 mL PBS (30 Bq). At each time point, three mice were sacrificed, the organs were collected and weighed, and the amount of radionuclide activity in tissues was measured using a gamma scintillation counter (PerkinElmer). The results are expressed as a percentage of injected activity per gram of tissue.

#### PET-CT Imaging and Biodistribution of ^64^Cu-10C6 MAb

For PET-CT imaging, three mice were injected in the tail vein with 10 MBq of ^64^Cu-10C6 (50 μg). Images were acquired 16 h after injection using a microPET-CT scanner (Inveon Siemens Medical Solutions) under anesthesia (isoflurane-O_2_). After imaging, the mice were sacrificed and biodistribution was performed.

### SPECT-CT Imaging on Experimental Dogs and Dogs With Spontaneous DLBCL

The SPECT-CT protocols applied to experimental dogs and dogs with spontaneous DLBCL were identical. This imaging was conducted according to WSAVA Global Guidelines (World Small Animal Veterinary Association) and the protocol study was validated by the CERVO Ethics Committee (Comité d'Ethique pour la Recherche clinique et épidémiologique Vétérinaire d'Oniris, Nantes, France; CERVO-2016-21-V). For imaging on sick privately owned dogs, the pet owner's informed consent was collected. The Mab content of endotoxin is within the range recommended for humans. The dogs were premedicated with methylprednisolone (Solu-Medrol® 20, PFIZER PFE France, 1 mg.kg^−1^ intravenously) and promethazine (Phenergan® 2.5%, UCB Pharma SA, 0.3 mg.kg^−1^ subcutaneously). The ^111^In-10C6 was co-injected with naked 10C6 MAb under anesthesia for 30 min through an intravenous saline line in a front leg. A variable amount of cold antibody ranging from 0 to 1.5 mg.kg^−1^ was used. The injected activity of ^111^In-10C6 was 3.7 MBq/kg of body weight. SPECT-CT was performed at 1 h and daily for 1 week for experimental dogs and at 48 h for sick dogs. SPECT-CT images were acquired on a SPECT-CT camera (Optima 640, GE Medical Systems) with a medium-energy general-purpose collimator (MEGP). The acquisition time was 30 s for each of the 60 projections. Energy windows (15% width) were centered on the two major peaks at 172 keV and 247 keV. The reconstruction was done using Xeleris software and the ordered subset expectation maximization (OSEM) algorithm with two iterations and 10 subsets. Images were corrected for attenuation, based on computed tomography (120 keV, 10 mA).

## Results

### Production and Purification of Canine CD22 Immunogen for Mice Immunization

**Figure 1:**
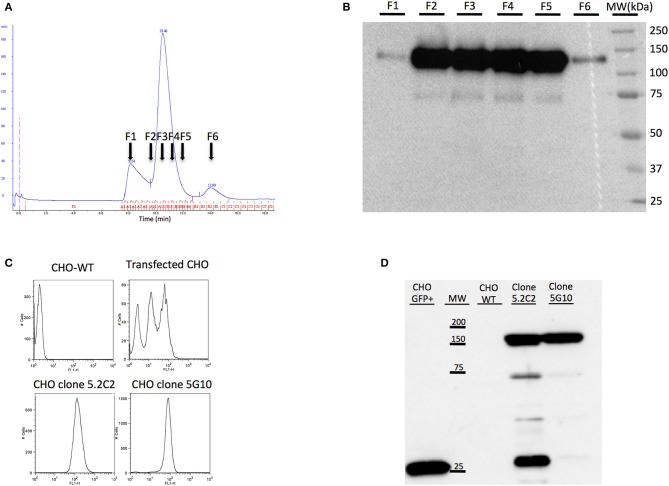
Production of soluble CD22c for mice immunization and production of CD22c expressing CHO cell line for hybridoma screening. **(A)** The recombinant canine CD22c-Beta2M protein produced by the transfected CHO cell clone showing the highest expression was purified by affinity chromatography using the 6H4 MAb followed by a Superdex 200 column. The results of size-exclusion chromatography show three distinct peaks. **(B)** Further analysis of fractions F1–F6 by Western blot showed high expression of CD22c-Beta2M in the major peak. **(C)** At the same time, CHO cells were transfected with a full-length CD22c merged to GFP (CD22c-GFP) for hybridoma screening using cytofluorometry. The two upper panels show FACS analysis of WT CHO (left) and polyclonal transfected CHO cells (right). The transfected CHO cells were further subcloned to select high-expressing clones: **CHO** 5C.2 and **CHO** 5G10 (bottom panels). **(D)** Western blot analysis allowed us to select clone **CHO** 5G10, which expresses a unique protein at the size corresponding to CD22cGFP for hybridoma screening.

A soluble form of canine CD22 usable for mice immunization and for monoclonal antibody characterization was produced by stable transfection into CHO cells. In its NH2 end this immunogen comprises the canine CD22 extracellular domain merged to the human beta-2-microglobulin with a peptide linker. Human β2m was chosen because of the availability in the laboratory of a specific anti-hβ2m monoclonal antibody (6H4) (23). This antibody enables the screening by ELISA of transfected cell supernatants for the production of the canine recombinant protein. One clone of transfected CHO cells (named 13E12) showing the strongest signal was amplified. One liter of 13E12 supernatant was produced and purified using HiTrap NHS-activated HP affinity column coupled to 6H4 antibody (HiTrap-6H4). The protein obtained after one-step affinity chromatography on HiTrap-6H4 was then purified by size-exclusion chromatography Superdex 200 column (Figure 1A). The purification fractions were analyzed by Western blot using the 6H4 antibody. The fractions corresponding to the major peak were further analyzed by Western blot. The molecular weight of the highlighted protein corresponds to the CD22-β2m fusion protein (Figure 1B). After purification, 2 mg of the fusion protein was obtained at sufficient purity for use as an immunogen. This amount was sufficient for the immunization of several mice and characterization of the MAbs produced.

### Production of a CHO Clone Expressing Canine CD22 at the Membrane for Hybridoma Screening

To screen hybridomas producing antibodies specific for canine CD22 using flow cytometry analysis, we produced CHO cells expressing membranous canine CD22. Since no antibodies specific for canine CD22 were available for the screening of transfected cells, the cytoplasmic domain of CD22 was merged with EGFP in order to be able to screen transfectants with flow cytometry. The polyclonal cell line was then cloned by limiting dilution and we selected the clones with the highest green fluorescence intensity, such as clones 5.2C2 and 5G10 (Figure 1C). Western blot analysis using an anti-EGFP antibody was performed on protein extracts from these clones and compared to CHO cells transfected with EGFP alone. A major band corresponding to the CD22c-EGFP molecular weight was highlighted for the 5G10 and 5.2C2 clones. However, an additional faint band (around 75,000 Da) and a strong band at lower molecular weight corresponding to EGFP alone were detected for the clone 5.2C2 (Figure 1D). The clone 5G10 that displayed the expected profile in Western blot was therefore selected for hybridoma supernatant screening.

### Immunization of Mice Against Canine CD22

**Figure 2:**
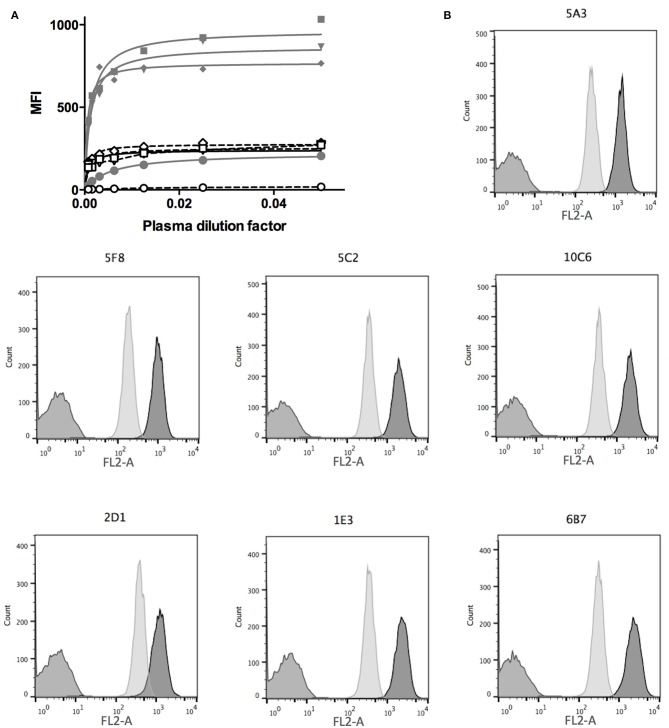
Mice immunization and hybridoma screening. **(A)** Mice were first injected with CD22c-Beta2M in complete Freund adjuvant at day 0 and 15 days later with the same amount of CD22c-Beta2M in incomplete Freund adjuvant. At day 29, the plasma of mice were harvested and used at serial dilutions in a FACS assay to label CD22-EGFP CHO clone 5G10 (gray lines, closed symbols) and the canine DLBCL cell-line CLBL-1 (dashed lines, open symbols) and compared to the signal obtained at the same dilution factors of the plasma before antigen injection (close and open circles). A clear signal increase at day 29 attests for immunization of mice. The lower signal obtained on CLBL-1 is consistent with a low CD22 expression on canine DLBCL. **(B)** The hybridomas obtained after fusion of splenocytes of an immunized mouse were screened by FACS analysis using CD22-EGFP CHO cells (dark gray) as a positive control, WT CHO cells as a negative control (medium gray). The specificity for the CD22 antigen was confirmed on the CLBL-1 cell line (light gray). Here are shown the results obtained for the seven hybridomas specific for CD22c, isolated from the fusion of the splenocytes of one mouse.

Three mice were immunized with the canine CD22c-β2m fusion protein. The serums of immunized mice were assessed for anti-CD22c antibody production by flow cytometry analysis using the CD22c-EGFP transfected CHO cell clone 5G10. The pre-immune serum at day 0, which was used as negative control, displayed a strong background on transfected CHO cells. However, at day 29 after the second antigen injection, significant labeling was obtained, attesting to the production of anti-CD22c by the mice immunized against the CD22c-β2m immunogen. It also validated the CD22c-EGFP transfected CHO cell clone 5G10 for the screening of hybridoma supernatant (Figure 2A). We further analyzed the serum of immunized mice using the canine diffuse large B-cell lymphoma (DLBCL) cell line CLBL-1. Because of the lack of available anti-CD22c antibody, we were not able to determine the expression level of this antigen on the canine DLBCL cells. Here we show that, as for the 5G10 clone, we detected a specific labeling of the CLBL-1 cell line at day 29 compared with the pre-immune serum (day 0) attesting, as expected, that CD22 is expressed by canine B-cell lymphoma and that the immunization with soluble CD22c-β2m is efficient at generating the production of antibodies able to bind the CD22c on its native form. Interestingly, the dilutions giving half of the maximum binding on the CD22c-EGFP transfected CHO cells and on CLBL-1 cells were very close (1:1,050 and 1:976, respectively, with the best immunized mouse), attesting that the anti-CD22 antibodies from the serum of immunized mouse recognized CD22c with the same affinity on both cell lines (Figure 2A). Even if the signal was specific, it was lower on CLBL-1 cells as compared to the 5G10 CHO clone. This is consistent with the low expression level of membrane CD22 described in human DLBCL (27). At the end of immunization process, the most potent immunized mouse was sacrificed, the spleen was harvested, and the splenocytes were fused with the mouse myeloma cell line SP2/0 in order to obtain hybridomas.

### Screening of Hybridoma Supernatant

Ten to 15 days after the fusion of splenocytes, 500 hybridomas were screened to evaluate their efficiency in producing the anti-canine CD22 antibodies. We used the WT CHO cell line as a negative control and the CD22c-EGFP+ 5G10 CHO clone and the CLBL-1 cell line as positive controls. Seven hybridomas out of the 500 tested allowed strong labeling of the CD22c-EGFP+ 5G10 CHO clone and no staining on WT CHO cells, as expected for antibodies specific for canine CD22. The ability of the seven hybridoma supernatants to bind the CLBL-1 cell line confirmed this specificity for CD22c (Figure 2B). We confirmed the low expression level of CD22c on CLBL-1 cells compared to CD22c-EGFP+ 5G10 CHO clone observed with the plasma of immunized mice. Positive hybridomas were then cloned by limiting dilution. Once these clones were established, a pilot production of supernatant was performed to obtain a sufficient amount of MAb by purification with protein G to define their isotypes and affinity for CD22c by flow cytometry analysis. Six of the seven antibodies (5A3, 6B7, 1E3, 10C6, 5C2, 5F8) displayed an IgG_1, κ_ isotype and the last, 2D1 MAb, is an IgG_2b, κ_. The dissociation constants (Kd) of the seven MAbs were between 3.88 1E-8 M and 1.21 1E-10 M (Table 1). These affinities are satisfactory for nuclear medicine applications targeting tumor antigen for imaging and therapy.

**Table 1:** Dissociation constants and isotypes of the seven MAbs specific for canine CD22.

	**2D1**	**5A3**	**6B7**	**1E3**	**10C6**	**5C2**	**5F8**
Kd (M)	8.47E-10	3.88E-08	2.23E-10	9.80E-10	1.21E-10	3.08E-10	5.61E-09
*Std. error*	*9.92E-11*	*9.90E-09*	*6.01E-11*	*1.35E-10*	*1.78E-11*	*3.61E-11*	*5.07E-10*
Isotype	IgG2b,k	IgG1,k	IgG1,k	IgG1,k	IgG1,k	IgG1,k	IgG1,k

*Italic values indicates standard error*.

### Epitope Mapping

**Figure 3:**
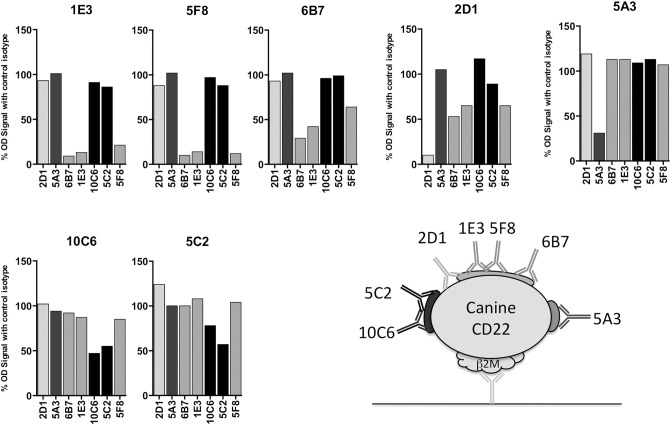
Epitope mapping of anti-CD22c antibodies. The seven MAbs selected for their specificity against the CD22c antigen were biotinylated and used in an ELISA assay using the CD22c-Beta2M for coating and the Beta2M antibody 6H4 for protein detection. Each biotinylated antibody was mixed with a ten-fold excess of each anti-CD22c clone, in order to identify the competing antibodies for the binding on CD22c. The results are expressed as the percentage of binding in comparison to the results obtained with a control isotype. The binding inhibition of each antibody by itself reflects the maximum inhibition of the test and constitutes a positive control of the competition test. A schematic representation of CD22c and the different epitopes recognized by the anti-CD22c MAbs is shown.

Apart from its use for mice immunization, the canine CD22-β2m fusion protein was also useful for characterizing the anti-canine CD22 antibodies. This construct was used in a sandwich ELISA assay to define an epitope mapping of the sites recognized on CD22 by the seven antibodies (Figure 3). For this purpose, each MAb was biotinylated and separately incubated with an excess of the seven anti-CD22c MAbs and a control isotype. Streptavidin HRP was used to detect antibody binding to CD22 in this competition assay regarding CD22 binding.

At least three different recognition patterns of CD22-β2m antibodies could be distinguished. The binding of the biotinylated 5A3 antibody on CD22-β2m was only inhibited by an excess of cold 5A3 MAb, indicating that it recognizes an epitope distinguishable from those recognized by the other MAbs on the CD22c molecule. The 10C6 and 5C2 antibodies recognized a single epitope, as indicated by their mutual binding inhibition. The antibodies 1E3, 5F8, and 6B7 also displayed a common pattern of cross inhibition for the binding on CD22c, indicating their specificity for a single epitope or overlapping epitopes. The 2D1 antibody displayed a more complex competition pattern: its binding was partially inhibited by 1E3, 5F8, and 6B7 antibodies, but 2D1 failed to impair 1E3, 5F8, and 6B7 binding to their own epitope. This could be consistent with a steric hindrance or in a non-mutually exclusive way with different conformational shapes of CD22 recognized by 2D1 and the 1E3, 5F8, and 6B7 group.

### IHC on Canine Lymph Node and Diffuse Large B-Cell Lymphomas

**Figure 4:**
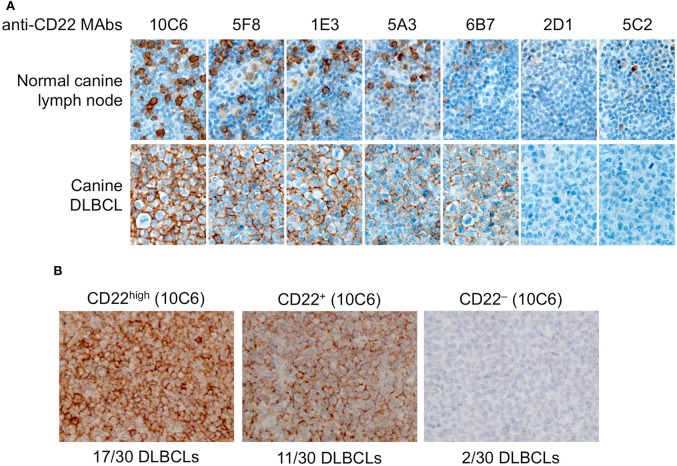
Immunohistochemistry to canine CD22 in a normal lymph node and in 30 canine diffuse large B-cell lymphomas. **(A)** In the normal canine lymph node (upper row), the 10C6, 5F8, and 1E3 MAbs labeled large B cells in germinal centers. CD22 was expressed at the membrane of most but not all B cells. The 5A3 and 6B7 MAbs had poor sensitivity and the 2D1 and 5C2 MAbs yielded no specific signal and were considered improper for IHC. In a CD22-positive canine DLBCL (lower row), the best IHC signal was obtained with the 10C6 clone. **(B)** In a series of 30 canine DLBCLs, 17 cases were strongly CD22-positive, 11 were moderately CD22-positive, and two were CD22-negative.

First we tested the seven antibodies in immunohistochemistry (IHC) for the detection of CD22c on formalin-fixed paraffin-embedded samples of normal lymph node. Whereas, all these antibodies were efficient at labeling CD22c in its native form on the CLBL-1 cell line by flow cytometry analysis (Figure 2B), they displayed strong differences in their ability to stain the CD22c antigen on histological sections, probably because of antigen denaturation after exposure to organic solvents during the process of histological preparation (Figure 4A). The clones 2D1 and 5C2 did not yield any specific staining of canine lymphocytes in the B-cell areas of normal lymph node and were therefore considered improper for immunohistochemistry. The clones 5A3 and 6B7 labeled sparse B cells in canine normal lymph nodes, whereas the clones 1E3, 5F8, and 10C6 were the most sensitive for IHC applications, because they labeled the membrane of large B cells in the germinal centers (Figure 4A), as well as the membrane of plasma cells (not shown). The 10C6 antibody clearly gave the best staining on healthy lymph node. Interestingly, even though 10C6 and 5C2 shared the same epitope on canine CD22 (Figure 3B), the 5C2 MAb was improper for IHC application, contrary to the former. This was also true for the 1E3 and 5F8 clones, which share a common epitope with the 6B7 MAb: the two former gave a satisfactory and comparable signal on CD22c in normal lymph node, while the 6B7 clone was inefficient in labeling this antigen on tissue sections. It is noteworthy that the distribution pattern of CD22-positive cells observed in the normal canine lymph node indicated that CD22 is not a pan-B-cell marker in dogs and is expressed by plasma cells.

Since these MAbs are the first anti-canine CD22c isolated to our knowledge, the CD22c expression status of canine DLBCLs was unknown at the beginning of the study. A canine DLBCL sample was immunolabeled with the seven anti-CD22c MAbs, showing that the 2D1 and 5C2 clones failed to react with lymphoma cells; the 5A3 and 6B7 clones gave a positive signal at the membrane of most neoplastic large B cells; and the 1E3, 5F8, and 10C6 MAbs positively labeled all of the neoplastic B cells, with the best IHC results obtained with the 10C6 clone (Figure 4A). From this IHC assay, we retained the 10C6 MAb for further development, for diagnosis in IHC, and for nuclear medicine applications, i.e., SPECT imaging and radioimmunotherapy.

We then proceeded to a first evaluation of CD22 expression on 30 canine DLBCLs by IHC, using the 10C6 MAb (Figure 4B). Among the 30 cases analyzed, 17 were strongly labeled with 10C6, 11 displayed intermediate CD22c membrane expression, and only two cases were negative for CD22 expression on DLBCL cells. There was no significant correlation between CD22 expression and the germinal-center or non-germinal-center phenotype of these cases. The high frequency of membrane CD22 expression by canine DLBCLs validates CD22 as a good antigen for targeted therapy of canine DLBCL, in accordance with what has been described in human DLBCL.

### Internalization Properties of Anti-CD22c MAbs

**Figure 5:**
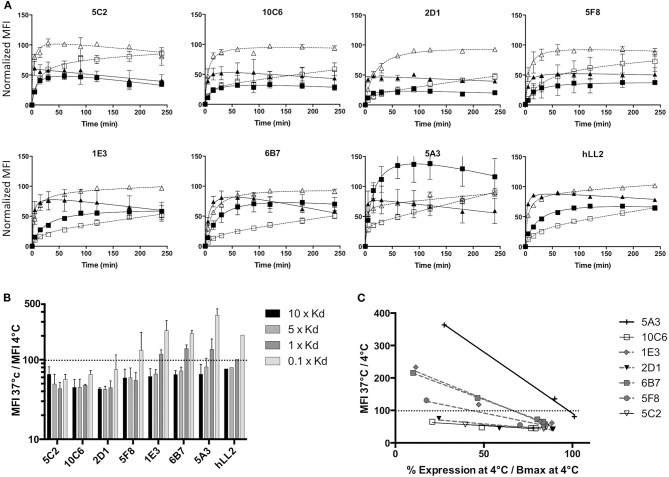
Internalization of anti-CD22c MAbs. The CLBL-1 cell line was incubated with each of the seven anti-CD22c MAbs at concentrations corresponding to 10-, 5-, 1-, and 0.1-fold their own dissociation constant at 4 and 37°C to evaluate MAb internalization at comparable levels of CLBL-1 CD22 saturation. **(A)** Kinetic follow-up of membrane CD22c expression at 4 and 37°C: at each kinetic time point, the cells were placed at 4°C, fixed with paraformaldehyde, labeled with an antimouse Ig Mab, and the MFI was measured by FACS. Open triangle, dashed line: 4°C, 10 × Kd; close triangle, solid line: 37°C, 10 × Kd; open square, dashed line: 4°C, 1 × Kd; closed square, solid line: 37°C, 1 × Kd. For comparison, the antihuman hLL2 antibody internalization was evaluated in the same conditions on the human Burkitt lymphoma cell line Daudi. Here are shown the mean results ± standard deviation of three independent assays. **(B)** Ratio of membrane CD22c expression at 37 vs. 4°C at 240 min for each concentration of anti-CD22c MAbs: the dashed line in this histogram corresponds to equal CD22 expression at 4 and 37°C. Whereas, clear internalization is observed for all antibodies at saturating concentrations of 10 × Kd and 5 × Kd (MFI 37°C/ MFI4°C <100), non-saturating amounts of antibody (1 × Kd and 0.1 × Kd) induced an over-expression of CD22 at the cell membrane when incubated with 1E3, 6B7, and 5A3 MAbs, and 5F8 to a lesser extent. **(C)** Ratio of CD22c expression at 37°C vs. 4°C as a function of the level of saturation of CD22c at 4°C for each MAb concentration: the dashed line corresponds to an equivalent CD22c expression at 37 and 4°C. Three groups of MAbs can be distinguished: highly internalizing antibodies 5C2, 10C6, and 2D1; intermediary internalizing antibodies 5F8, 1E3, and 6B7; and weakly internalizing antibody 5A3.

A characteristic of antibodies directed against human CD22 is their ability to internalize once bound to the antigen. This is of particular interest in the context of phenotypic imaging or radioimmunotherapy, because radiolabeled antibodies with a residualizing property would make it possible to sequester radioactivity within cell compartments after internalization, allowing higher activity deposition than a simpler membrane binding of the radiolabeled antibody (28). We thus determined the internalization ability of our CD22c-specific MAbs on the canine DLBCL CLBL-1 cell line, which naturally expresses CD22. Usually, CD22 internalization is evaluated on cells preloaded with saturating amounts of anti-CD22 MAb at 4°C to avoid membrane turnover. After washing, cells are placed at 37°C and internalization is evaluated at different time points. However, in nuclear medicine applications, DLBCL cells targeted by anti-CD22 antibodies are exposed to variable circulating antibody concentrations in the course of treatment, due to the mobilization of antibodies on healthy B cells and DLBCL cells, and due to antibody catabolism that progressively decreases the concentration of MAb in blood. We therefore wondered what the antibody binding to membrane CD22 antigen could be at various concentrations at 37°C compared to 4°C. Four MAb concentrations corresponding to 10-, 5-, 1-, and 0.1-fold their Kd value were used to compare the binding of the different anti-CD22 antibodies at comparable levels of antigen saturation. At different time points after the beginning of the incubation, the cells were placed at 4°C, washed with ice-cold PBS and labeled with an anti-mouse antibody to measure the mean fluorescence at each time point using flow cytometry. The membrane expression level of each antibody at 10- and 1-fold its Kd is shown in Figure 5A. A clear internalization of all MAbs is observed at 37°C with the antibody concentration corresponding to 10-fold the antibody Kd (saturation). However, a lower concentration corresponding to the Kd (half-saturation) results in higher membrane expression at 37°C than at 4°C of MAbs 1E3, 6B7, and 5A3, indicates that some antibodies may in some instances stabilize CD22 at the cell membrane, at least for the duration of the assay (6 h). The relative expression level at 37 vs. 4°C at the four concentrations investigated in this experiment is summarized in Figure 5B. Only the MAbs 5C2, 10C6, and 2D1 were able to internalize at low concentration (0.1-fold the MAb Kd), indicating that internalization not only depends on the antibody binding on its target, but also on the density of bound antibody at the cell surface. Indeed, the internalizations of the different antibodies were variously affected by saturation level, as shown in Figure 5C where the percentage of expression at 37°C relative to 4°C is plotted against the percentage of saturation at 4°C observed at each concentration used in the test. This graph emphasizes that a threshold of antigen occupation needs to be reached for internalization to take place. This threshold was low for 5C2, 10C6, and 2D1 because they were internalized at the lowest MAb concentrations used in the test. Conversely, internalization was observed for MAbs 1E3, 6B7, and 5F8 when the surface density reached 45–65% saturation and even more for the 5A3 MAb. It is puzzling that these strong, intermediate, and low internalizing capabilities segregate with the epitopes recognized, respectively, by 10C6 and 5C2; 6B7, 1E3, and 5F8; and 5A3 MAbs. One could hypothesize that the topology of MAb binding to CD22 affects the efficiency of the internalization process.

Finally, we evaluated the internalization property on human lymphoma cell line of the anti-Human CD22 hLL-2 MAb, already used in phase I/II clinical trials for DLBCL treatment in humans, to obtain an element of comparison with the internalization property of the anti-CD22c MAbs. The internalization profile of hLL-2 MAb at different concentrations was similar to the profile of 6B7 or 1E3 MAbs. As for the 6B7 and 1E3 MAbs, membrane stabilization of CD22 was observed for concentrations corresponding to 0.1- and 1-fold the dissociation constant of the antibody. This indicates that our antibodies against CD22c have similar properties as compared to the anti-CD22h hLL-2, and thus may be suitable to perform preclinical trials in dogs with spontaneous DLBCL for new imaging and therapeutic protocol testing.

### Biodistribution of Anti-CD22c in Mice Xenografted With CLBL-1

**Figure 6:**
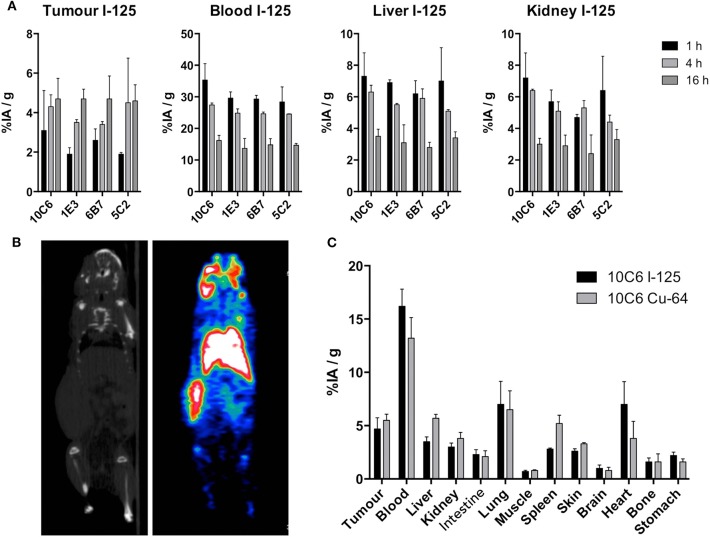
Biodistribution of anti-CD22 antibodies and PET imaging of CLBL-1 xenografts in nude mice. **(A)** Biodistribution to tumors, blood, liver, and kidney 1, 4, and 16 h after injection of 6.10^−6^ g of 10C6, 1E3, 5C2, and 6B7 radiolabeled with ^125^I. Here are shown the mean results ± standard deviation obtained on three mice. **(B)** PET-CT imaging 16 h after injection of ^64^Cu-10C6. The CLBL-1 cell line was engrafted in the left flank of nude mice. Left panel: CT scan of the imaged mouse. Right panel: PET imaging showing the radiolabeled tumor in the left flank of the nude mouse. **(C)** Comparison between the biodistribution of 10C6 radiolabeled with ^125^I and ^64^Cu. Immediately after imaging at 16 h post-injection of the ^64^Cu-radiolabeled antibody, the mouse was sacrificed and the biodistribution in the different organs of interest was evaluated and compared to the biodistribution of ^125^I-10C6 at the same time point.

Because of the different internalizing behaviors of the anti-cCD22 antibodies, we wondered which one would be the most appropriate MAb for imaging and radioimmunotherapy. We chose to compare 10C6, 5C2, 6B7, and 1E3, which displayed distinct internalization patterns and high affinities, in a biodistribution assay. The 2D1 and 5A3 MAbs were excluded from this assay because of the IgG2b isotype of 2D1 and the low affinity of 5A3. Eight-week-old NMRI-nu mice were subcutaneously engrafted in the flank with five million CLBL-1 cells. Fourteen days after engraftment, MAbs 10C6, 5C2, 6B7, and 1E3 radiolabeled with ^125^I were injected in the tail vein. Mice were sacrificed at 1, 4, and 16 h after injection and the organs were weighed and counted in a gamma counter. The results of biodistribution are shown in Figure 6. No significant differences of accumulated activity in tumors and healthy organs could be objectified between the four antibodies. The cumulated activity in the tumor 16 h after injection reached around 4.5% IA/g with the four antibodies tested. Although the activity to the tumor was quite low, the increase over time of the cumulated activity confirmed the specificity of canine CD22 targeting in this mouse model. For imaging and radioimmunotherapy applications, high-affinity antibodies were favored; 5C2 and 10C6 MAbs were the anti-CD22c clones with the highest affinities in our antibody panel, but 10C6 proved to be usable for diagnosis of spontaneous canine DLBCL, contrary to 5C2, which was inefficient for IHC (Figure 4A). We therefore pursued our investigations with the 10C6 antibody to validate its value for further applications in veterinary medicine.

### PET Imaging in Mice Xenografted With CLBL-1

Our primary goal, isolating anti-CD22c MAbs, was to undertake imaging and radioimmunotherapy assays in dogs with spontaneous DLBCL. We wished to perform SPECT-CT with indium-111 radiolabeled antibody and radioimmunotherapy with yttrium-90. This requires the coupling of a chelating agent, enabling radiolabeling with these isotopes. We sought to ensure that the modification of the 10C6 antibody by the chelating agent DOTA-NCS did not affect its ability to bind to the CD22 antigen. The 10C6 MAb was coupled to DOTA using p-SCN-Bn-DOTA precursors able to covalently link to the lysine side chain on the antibody. The mean number of DOTA chelators on the antibody was estimated by determining the radiolabeling yield of the 10C6 MAb radiolabeled with increasing amounts of copper-64 using thin-layer chromatography. A mean number of 2.78 DOTA per antibody was calculated, which is adapted for nuclear medicine applications. ^64^Cu-10C6 was then used in PET-CT imaging of nude mice 14 days after engraftment with the CLBL-1 cell line. PET-CT images were acquired 16 h after injection of 10 MBq of ^64^Cu-10C6 (Figure 6B). Mice were then sacrificed and the biodistribution of ^64^Cu-10C6 was evaluated on tumor and healthy organs (Figure 6A). PET imaging provides clear visualization of the subcutaneous CLBL-1 xenograft in the right flank. The intense staining of the liver and the lungs with ^64^Cu-10C6 MAb is based on the large volume of these organs and their high blood contents. Although copper catabolism implies liver and biliary excretion, we only detected a slight increase in the cumulated activity of ^64^Cu-10C6 MAb compared to ^125^I-10C6 to the liver (5.46 ± 0.35 vs. 3.55 ± 0.44% IA/g, respectively). This is consistent with the higher activity to the blood for ^125^I-10C6 compared to ^64^Cu-10C6. After cellular catabolism of the antibody, iodine is released as an iodo-tyrosine in the interstitial space. In contrast, chelated metallic isotopes display residualizing properties leading to their sequestration within the cell compartment. This residualizing property may explain in part the higher activity measured to the spleen with ^64^Cu-10C6 as compared to ^125^I-10C6 (5.24 ± 0.35 vs. 2.83 ± 0.1%IA/g for ^125^I-10C6), and the cumulated activity uptake to in the tumor that was also slightly higher with ^64^Cu-10C6 MAb as compared to ^125^I-10C6 (5.45 ± 0.57 vs. 4.70 ± 1.04%IA/g, respectively). These results indicate that 10C6 can be efficiently radiolabeled with satisfactory stability, either by iodination or with a metallic isotope using the 10C6-DOTA MAb. The capacity of 10C6 antibody to specifically label canine DLBCL *in vivo* in mouse xenograft models validates its usefulness for future clinical assays in dogs with spontaneous DLBCL for diagnosis, imaging, and radioimmunotherapy.

### SPECT-CT Imaging With ^111^In-10C6 MAb on Experimental Dogs

**Figure 7:**
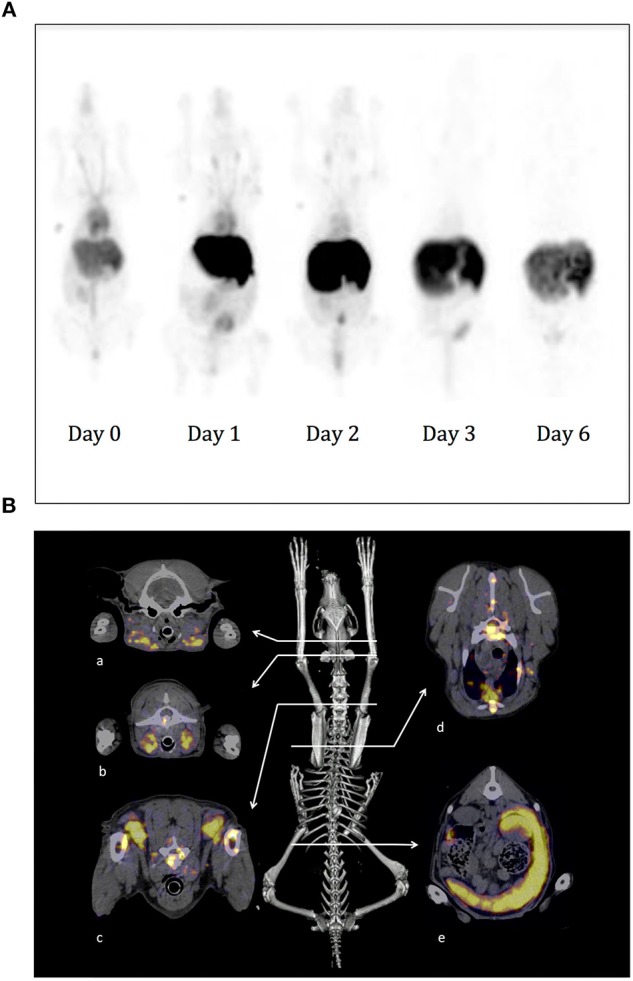
SPECT-CT imaging using ^111^In-10C6 MAb on experimental and sick dogs. **(A)** A healthy beagle weighing 11.5 kg was injected under anesthesia with ^111^In-10C6 at a total activity of 3.7 MBq/Kg and a co-injection of 1.5 mg/kg of cold 10C6. Images were acquired 1 h after injection and then at day 1, 2, 3, and 6. Here are shown a planar projection of the whole-body SPECT images. The nose of the dog and the liver, which are highly vascularized in dogs, appear with a strong contrast. The vasculature is visible up to day 2. The signal in the abdomen corresponds to the elimination of radioactivity in the feces after clearance of ^111^In-10C6 by the hepatobiliary system. In the first three images, 1 ml of blood taken at the beginning of acquisition positioned close to the anterior left leg was imaged in order to obtain a reference of blood activity. **(B)** SPECT-CT images of a dog with spontaneous DLBCL diagnosed at the veterinary hospital. The dog was a 4-year-old female Flat-coated Retriever with stage V multicentric lymphoma. SPECT-CT acquisition was performed 45 h after radiopharmaceutical injection (0.75 mg ^111^In-10C6; specific activity 113.9 MBq.mg^−1^; without co-injection of cold 10C6). The skeleton is shown as the anatomic landmark. Here are shown the fusion SPECT and CT images where tumor sites could be easily visualized. a: Mandibular lymph nodes; b: retropharyngeal lymph nodes; c: prescapular lymph nodes; d: body of a thoracic vertebra, substernal lymph node; e: spleen.

Before proceeding to imaging on sick dogs, we wished to evaluate the biodistribution of the ^111^In-10C6 MAb on experimental dogs. The protocol applied to experimental dogs was the same as that planned for dogs with spontaneous DLBCL. The Mab content of endotoxin was tested and was within the range recommended for humans. Given that we used murine antibodies, the dogs were premedicated with methylprednisolone and promethazine in order to avoid any anaphylactic shock at the time of injection. Fourteen dogs were imaged with this protocol without adverse effect, thus validating the safety of this radiolabeled Mab. The objectives of repeating images on several dogs at different time points were to obtain a set of images allowing the implementation of a population pharmacokinetics and to estimate the doses at healthy organs using quantitative imaging. We also took advantage of this possibility to take images on experimental dogs to test the consequences on the biodistribution and pharmacokinetics of cold antibody co-injection with ^111^In-10C6 MAb since no specific study has addressed this issue in humans (manuscript in preparation). More generally, this set of images was useful in illustrating the particularity of Mab distribution in dogs compared to humans to facilitate the interpretation of images at diagnosis on sick dogs. Images of the SPECT signal detected on an experimental dog are shown in Figure 7A. The best contrast images were obtained at 2 days after injection. The more salient differences with humans were a stronger signal on the dog's nose and liver. Although we cannot exclude a specific binding of the antibody on these sites, this enhanced uptake in dogs compared to the corresponding area in human anatomy may also result from a higher vascularization in dogs. This specific feature in dogs will most particularly lead to higher dose deposition on the liver. However, preliminary evaluation of the dose deposition on the liver of dogs indicates that it remains under the toxic threshold for injected activity within the range of the therapeutic dose evaluated in humans (manuscript in preparation). We therefore proceed to the imaging of dogs with spontaneous DLBCL.

### SPECT-CT Imaging With ^111^In-10C6 MAb on Dogs With Spontaneous DLBCL

Three dogs diagnosed at a veterinary hospital for DLBCL were enrolled for SPECT-CT imaging. ^111^In-10C6 MAb was injected according to the protocol applied for experimental dogs with no adverse effect. In each of the three cases, we clearly observed tumor sites already detected at initial diagnose and an additional tumor that was undetectable with classical diagnostic methods.

Figure 7B provides an example of a SPECT-CT image performed on a dog with spontaneous DLBCL. The enrolled dog was a 4-year-old female Flat-coated Retriever with stage V multicentric lymphoma. The physical examination revealed apathy, weight loss (weight at diagnosis, 25 kg), dysorexia, hyperthermia, generalized lymphadenopathy (peripheral, thoracic, and abdominal), and abdominal ultrasound showed splenic infiltration. A myelogram on red marrow demonstrated the presence of a contingent of atypical cells, medium to large in size, with a high nucleocytoplasmic ratio. Their morphology was similar to that of cells invading lymph nodes and that of circulating atypical lymphoid cells. The 10C6 Mab was used for immunohistochemistry (IHC) performed on a biopsy of the left prescapular lymph node. The lymph node is invaded by cells with strong membrane labeling. Based on this IHC and histologic feature, it was possible to establish a diagnosis of DLBCL with CD22 overexpression as shown. SPECT-CT acquisition was performed 45 h after radiopharmaceutical injection (0.75 mg ^111^In-10C6; specific activity 113.9 MBq.mg^−1^). Radiopharmaceutical injection was well-tolerated. Lymphadenopathy was demonstrated on SPECT-CT images showing tumoral infiltration of the spleen and numerous lymph nodes, as shown for retropharyngeal lymph nodes whose status had remained undetermined until then (Figure 7B). In accordance with the results of the myelogram, SPECT-CT confirmed the medullary invasion that was then qualified as severe.

## Discussion

Here we describe a simple method to generate monoclonal antibodies against all types of membrane proteins with a single transmembrane domain. The possibility of expressing these antigens after stable transfection in CHO cells as a soluble form merged to a tag (β2m herein) enables to easily identify productive CHO clones using an anti-tag antibody in a one step ELISA test. This anti-tag antibody coupled to an affinity chromatography column also enables, after a one-step purification process, to purify sufficient amounts of recombinant protein to immunize mice. This soluble antigen is also useful as a reagent in binding assays to perform immunoreactivity quality controls of the MAbs once radiolabeled. Because we wanted to use this antigen for nuclear medicine applications, we chose to perform hybridoma supernatant screening by flow cytometry analysis in order to discard any antibodies that would recognize epitopes on the unfold antigen in ELISA test. Furthermore, since soluble CD22c was merged to the human β2m, some hybridomas generated against β2m will not recognize CD22-transfected CHO cells. This makes it possible to rapidly perform a first screening of hybridomas producing antibodies recognizing CD22-transfected CHO cells, which obviates the need for a preliminary ELISA with human β2m used as a negative control. This screening method enabled us to screen around 500 hybridoma supernatants and resulted in the isolation of seven monoclonal antibodies reactive against CD22c in its native conformation expressed by the canine DLBCL cell line CLBL-1. The isolation of numerous MAbs with the same antigen specificity gave the opportunity to select the most adapted one for *in vivo* imaging and radioimmunotherapy applications and for diagnosis using immunohistochemistry. At least three distinct epitopes on CD22c antigen were defined in our competition assay with the seven anti-CD22c MAbs. Antibodies 5C2 and 10C6 or 1E3 and 5F8 displayed very similar competition patterns. It is likely that each antibody represents distinct clones owing to their differences in affinity or reactivity by IHC assay: 10C6 gave the best results in IHC, contrary to 5C2, which failed to detect CD22 despite a shared recognition pattern and close dissociation constant values.

A hallmark of CD22 is its clathrin-dependent endocytosis upon antibody binding (29). In the specific context of CD22 targeting in nuclear medicine, the intracellular behavior of the radiopharmaceutical is of primary importance, since it impacts the dose deposition and thus the quality of phenotypic imaging and the efficacy of RIT. Internalized isotopes with a residualizing property like chelated metallic isotopes are trapped within the cell after vector catabolism, enhancing the dose deposition, while iodinated antibody catabolism produces iodo-tyrosine, which is rapidly excreted from the cell (30, 31). This internalization property also determines the efficacy of antibody-drug conjugates (ADC) as it largely determines the efficiency of intracellular release of the drug linked to an antibody. The fate of antibody bound on CD22 once internalized has been largely investigated but remains controversial, some authors favoring its routing in a recycling pathway back to the cell surface via recycling endosome while others arguing for a routing to degradation in lysosome (32, 33). Most often, the CD22 internalization process was evaluated with one antibody on several B-cell lymphomas, underlining the variable internalization capabilities of cell lines of different origins.

Here we sought to take advantage of our panel of antibodies to investigate the variability of the internalization process depending on the antibodies. Internalization was evaluated by measuring antibody binding at 4 and 37°C using the CLBL-1 cell line at different antibody concentrations corresponding to comparable CD22 antigen saturation levels for all antibodies. It clearly appears that at saturating concentration [10-fold the dissociation constant (Kd) of the MAb], internalization was observed for all antibodies with differences in kinetics and intensity. However, at a non-saturating concentration, salient differences appear between antibodies. Some of them, such as 5C2, 10C6, and 2D1, retain their ability to internalize, while other MAbs such as 1E3, 5F8, and 6B7 and above all 5C3 are stabilized at the cell surface, resulting in a higher membrane expression at 37°C than at 4°C. This would indicate that internalization not only depends on antibody binding on its target but also on the density of bound antibody at the cell surface. A threshold of antigen occupation, which is characteristic of each antibody, needs to be surpassed for internalization to take place during the 6 h of the assay. Interestingly, these differences in internalization properties segregated quite well with the epitope recognized on the CD22c antigen. However, the 2D1 antibody is an exception and recognizes an epitope overlapping with 5F8, 1E3, and 6B7 antibodies but displays a very different internalization profile.

The different abilities of monovalent and divalent antibody fragment or native antibody to induce internalization of the transferrin receptor (TfR)—usually taken as a paradigm of clathrin-dependent internalization and recycling to the membrane—has already been investigated. Saturation of transferrin receptor with the monovalent F(ab)′ fragment had no effect on TfR internalization, contrary to F(ab)′2 and IgG (34). We hypothesize that the ability of each antibody to crosslink two CD22c antigens can be either energetically favorable or unfavorable depending on the topology of the antibody–antigen (epitope–paratope) interaction. In the case of 10C6 and 5C2 antibodies, the binding of the first valence of the antibody to CD22c might promote the binding of the second valence, allowing for crosslinking of surface CD22c and internalization at a non-saturating antibody concentration. Conversely, in the case of 1E3, 5F8, 6B7, and 5C3 MAbs, a high antibody burden would be required to offset the energetically unfavorable crosslinking of CD22c in order it occurs at a frequency and/or for a sufficient time course required for initiation of the CD22 internalization. The objective of this article was not to dissect in detail the mechanism and the regulation of CD22 internalization. However, because we were able to distinguish high, intermediate and low internalizing anti-CD22c MAbs, we wondered if this property could modify the dose deposition to cancer cells within a RIT assay.

To address this question, biodistributions were performed with 5C2 and 10C6 on the one hand and 1E3 and 6B7 MAbs on the other hand, which display high and medium internalization abilities, respectively. For this purpose, nude mice were engrafted subcutaneously with CLBL-1 cells and injected 2 weeks after engraftment with each of the four MAbs radiolabeled with ^125^I. Even if the activity to the tumor remains rather low, these results are consistent with what is described in the literature after injection of anti-CD22 MAb hLL2 in nude mice bearing human lymphoma xenografts (28). No significant differences were observed regarding the biodistribution of the different MAbs tested in healthy organs. Kidney and liver activities could be explained by the blood kinetics and appeared consistent with an absence of antibody binding in these organs, as expected with mouse IgG_1_ specific for a xenogeneic antigen. The tumor uptake of the radiolabeled MAb is also similar for the four MAbs tested. The only notable but non-significant difference is a trend for 10C6 and 5C2 antibodies to accumulate more rapidly in the tumor. As an example, 4 h post-injection the activity into the tumor for the 10C6 antibody was close to what was observed at 16 h (4.3 ± 0.6 and 4.7 ± 1.0%IA/g, respectively), contrary to 6B7 (3.4 ± 0.2 and 4.7 ± 1.2%IA/g, respectively). Since 10C6 is the MAb with highest affinity and the best ability to stain CD22c by immunohistochemistry for spontaneous canine DLBCL diagnosis, we wished to further evaluate it as a potent radiopharmaceutical to perform phenotypic imaging and RIT. These applications require using the 10C6 MAb radiolabeled with metallic isotopes, as ^111^In for SPECT-CT imaging, ^64^Cu for PET-CT imaging, and ^90^Y or ^177^Lu for RIT. NCS-DOTA could be used as a bifunctional chelator to label immunoconjugates with these three different isotopes. It was thus necessary to check that the coupling of the chelating agent did not affect antibody affinity. The covalent linking of the bi-functional chelating agent NCS-DOTA on the side chains of lysine could in some instances be detrimental to the reactivity of the antibody due to steric hindrance when lysine participates in the recognition site of the antibody or when lysine is close to the recognition site. Modifying an antibody with a bi-functional chelating agent can also modify its pharmacokinetics depending on the mean number of chelating agents coupled to the antibody. These immune-reactivity and pharmacokinetic parameters should be tested for each monoclonal antibody since each of them displays a unique sequence with variable numbers of lysine in their variable regions.

To this end, the 10C6 MAb was coupled to DOTA and radiolabeled with the positron emitter ^64^Cu in order to perform positron emission tomography (PET-CT) imaging. Despite the low level of antibody accumulation using this tumor model, we were able to clearly detect subcutaneous CLBL-1 tumor 16 h after ^64^Cu-10C6 injections, validating that the 10C6 antibody affinity is preserved after coupling with DOTA. Mice were sacrificed after imaging and the biodistribution of ^64^Cu-10C6 was evaluated. The activity to the tumor was comparable to what was observed with the iodinated MAb. The highest activity to the heart observed with the iodinated antibody was consistent with a higher activity in the blood compared to ^64^Cu-10C6. In addition, the highest activity within the liver was expected due to liver elimination of copper. We used ^64^Cu in this assay because of the availability of a micro PET-CT device for imaging on mice. We are aware, however, that DOTA is not the best chelating agent for copper (35). Although the ^64^Cu-10C6 was purified from free ^64^Cu after radiolabeling, free copper can still be released into the blood flow and accumulate in the organs responsible for copper clearance and elimination. This may explain the high liver uptake noted 16 h after injection, the faster decay in blood and the poor increase in tumor uptake that would be expected with a residualizing metallic isotope compared to ^125^I-10C6. For future applications in dogs, we plan to use the DOTA chelating agent to radiolabel 10C6 MAb with ^111^In for SPECT-CT imaging and with ^90^Y for RIT. These isotopes are more stably chelated with DOTA than copper. This may enable a higher dose deposition to the tumor, as shown by Sharkey et al. comparing iodinate and indium-labeled antibody biodistribution targeting CD22 on nude mice bearing the human B-cell NHL RL cell-line (36).

At the end of this *in vitro* and *in vivo* evaluation we selected 10C6 Mab for IHC and SPECT-CT imaging. First of all, we used the experimental dog to validate the imaging protocol and determine the binding property of ^111^In-10C6 on healthy organs. We observed a strong antibody accretion on the liver compared to humans. Several explanations could account for this high liver content. The recycling of mouse IgG via dog FcRn binding may be inefficient, as mentioned by Bergson et al. (37), entailing a rapid degradation of antibody after endocytosis and clearance via the hepatobiliary route. Anatomical differences between humans and dogs could also explain differential antibody accretion. The high vascularization of the dog nose compared to humans also results in a stronger signal in the former species. This is also true for liver, which is proportionally larger in dogs than in humans and therefore mobilizes a higher proportion of the total blood content. This needs to be taken into account for toxicity concerns in the course of the RIT planned, especially for the highest dose plan to be evaluated in a dose escalation. However, a preliminary evaluation indicates that the doses to liver remain at a level allowing injection of activity within the range of the therapeutic window determined in humans (manuscript in preparation).

These results prompt us to use ^111^In-10C6 on dogs with spontaneous DLBCL in order to validate the ability of this antibody to target tumor sites. Three sick dogs were imaged. We were able to visualize the tumor site diagnosed by classical examination using SPECT-CT imaging. In addition, SPECT-CT made it possible to clearly visualize the tumor site not detected by current examination techniques, such as the bone marrow invasion in the example provided in this article. Although myelogram analysis indicated the presence of tumor cells in the marrow, the extent of the tumor invasion could not be assessed. The exclusion criterion of 20% of bone marrow invasion that we retain for future RIT assays could only be assessed with imaging. SPECT-CT could therefore be useful for the staging of the pathology, although ^18^FDG could be used to this end. Another input of SPECT-CT imaging not provided by ^18^FDG is that it can provide quantitative imaging and dosimetry, making it possible to extrapolate the actual dose to healthy organs and tumor sites in the course of RIT treatment. The next step of our project will be to perform the dosimetry analysis on experimental dogs in order to determine a population pharmacokinetic model that can be used in the clinical context to evaluate, on a limited number of images of sick dogs, whether they conform to the general pharmacokinetic model or if discrepancies would require adjusting the injected activity to avoid toxicity or to reach a therapeutic dose to tumor cells. We plan to use this antibody to treat dogs with spontaneous DLBCL based on the encouraging results previously obtained on human DLBCL in a phase I/II assay. This clinical trial in dogs with a preclinical value for human patients could ensure more accurate evaluation of the relevance of CD22 targeting for DLBCL management. It also ensures the transfer of new methods in quantitative imaging and new therapeutic approaches, which are difficult to evaluate in humans, to the clinic.

## Data Availability Statement

All datasets generated for this study are included in the article/supplementary material.

## Ethics Statement

The animal study was reviewed and approved by Ethics Committee of Pays de la Loire. Written informed consent was obtained from the owners for the participation of their animals in this study.

## Author Contributions

FD participated in hybridoma production, antibody radiolabeling, biodistribution, and imaging studies. He was the major contributor in writing the manuscript. MBe, KB, and MD isolated and produced naked and radiolabeled antibodies. FN determined their ability to be used in immunohistochemistry and performed IHC on dog biopsy. JA provided the canine tumor tissue bank and contributed to the development of the project. FE characterized MAb internalization property, performed epitope mapping, and participated to biodistribution on mouse model. She supervised the logistics for dog inclusion in the assay and the clinical assistance at the Veterinary Teaching Hospital. VG-G participated to the assays of MAb internalization. CM supervised and executed experiments on rodents. CI participated in dog imaging. AV radiolabeled 10C6 Mab with Indium-111. MBo radiolabeled 10C6 MAb with copper-64. NC performed SPECT-CT acquisitions. CB-M interpreted SPECT-CT images. All authors read and approved the final manuscript. In addition to the actual implementation of automated radiolabeling in the annex of the hospital pharmacy, AV provided all the necessary information for the writing of this manuscript and participated in the preliminary discussion concerning the process and organization of the radiolabeling of 10C6 antibody for the clinical trial on dogs with spontaneous DLBCL.

### Conflict of Interest

The authors declare that the research was conducted in the absence of any commercial or financial relationships that could be construed as a potential conflict of interest.

## References

[1] Casadei B Pellegrini C Pulsoni A Annechini G De Renzo A Stefoni V. 90-yttrium-ibritumomab tiuxetan consolidation of fludarabine, mitoxantrone, rituximab in intermediate/high-risk follicular lymphoma: updated long-term results after a median follow-up of 7 years. Cancer Med. (2016) 5:1093–7. 10.1002/cam4.684 26990782 PMC4924367

[2] Morschhauser F Radford J Van Hoof A Vitolo U Soubeyran P Tilly H. Phase III trial of consolidation therapy with yttrium-90-ibritumomab tiuxetan compared with no additional therapy after first remission in advanced follicular lymphoma. J Clin Oncol. (2008) 26:5156–64. 10.1200/JCO.2008.17.2015 18854568

[3] Rizzieri D. Zevalin((R)) (ibritumomab tiuxetan): after more than a decade of treatment experience, what have we learned? Crit Rev Oncol Hematol. (2016) 105:5–17. 10.1016/j.critrevonc.2016.07.008 27497027

[4] Tomblyn M. Radioimmunotherapy for B-cell non-hodgkin lymphomas. Cancer Control. (2012) 19:196–203. 10.1177/107327481201900304 22710895

[5] Kaminski MS Zelenetz AD Press OW Saleh M Leonard J Fehrenbacher L Pivotal study of iodine I 131 tositumomab for chemotherapy-refractory low-grade or transformed low-grade B-cell non-Hodgkin's lymphomas. J Clin Oncol. (2001) 19:3918–28. 10.1200/JCO.2001.19.19.3918 11579112

[6] Scholz CW Pinto A Linkesch W Linden O Viardot A Keller U. (90)Yttrium-ibritumomab-tiuxetan as first-line treatment for follicular lymphoma: 30 months of follow-up data from an international multicenter phase II clinical trial. J Clin Oncol. (2013) 31:308–13. 10.1200/JCO.2011.41.1553 23233718

[7] Ferrucci PF Vanazzi A Grana CM Cremonesi M Bartolomei M Chinol M. High activity 90Y-ibritumomab tiuxetan (Zevalin) with peripheral blood progenitor cells support in patients with refractory/resistant B-cell non-Hodgkin lymphomas. Br J Haematol. (2007) 139:590–9. 10.1111/j.1365-2141.2007.06869.x 17979944

[8] Gopal AK Rajendran JG Gooley TA Pagel JM Fisher DR Petersdorf SH High-dose [131I]tositumomab (anti-CD20) radioimmunotherapy and autologous hematopoietic stem-cell transplantation for adults > or = 60 years old with relapsed or refractory B-cell lymphoma. J Clin Oncol. (2007) 25:1396–402. 10.1200/JCO.2006.09.1215 17312330

[9] Illidge TM Bayne M Brown NS Chilton S Cragg MS Glennie MJ. Phase 1/2 study of fractionated (131)I-rituximab in low-grade B-cell lymphoma: the effect of prior rituximab dosing and tumor burden on subsequent radioimmunotherapy. Blood. (2009) 113:1412–21. 10.1182/blood-2008-08-175653 19074729

[10] Illidge TM Mayes S Pettengell R Bates AT Bayne M Radford JA Fractionated (90)Y-ibritumomab tiuxetan radioimmunotherapy as an initial therapy of follicular lymphoma: an international phase II study in patients requiring treatment according to GELF/BNLI criteria. J Clin Oncol. (2014) 32:212–8. 10.1200/JCO.2013.50.3110 24297953

[11] Hoelzer D. Novel antibody-based therapies for acute lymphoblastic leukemia. Hematology Am Soc Hematol Educ Program. (2011) 2011:243–9. 10.1182/asheducation-2011.1.243 22160041

[12] Juweid ME. Radioimmunotherapy of B-cell non-Hodgkin's lymphoma: from clinical trials to clinical practice. J Nucl Med. (2002) 43:1507–29. 12411555

[13] Chevallier P Eugene T Robillard N Isnard F Nicolini F Escoffre-Barbe M. (90)Y-labelled anti-CD22 epratuzumab tetraxetan in adults with refractory or relapsed CD22-positive B-cell acute lymphoblastic leukaemia: a phase 1 dose-escalation study. Lancet Haematol. (2015) 2:e108–17. 10.1016/S2352-3026(15)00020-4 26687796

[14] Kraeber-Bodere F Pallardy A Maisonneuve H Campion L Moreau A Soubeyran I Consolidation anti-CD22 fractionated radioimmunotherapy with (90)Y-epratuzumab tetraxetan following R-CHOP in elderly patients with diffuse large B-cell lymphoma: a prospective, single group, phase 2 trial. Lancet Haematol. (2017) 4:e45 10.1016/S2352-3026(16)30168-5 27964867

[15] Macor P Secco E Zorzet S Tripodo C Celeghini C Tedesco F. An update on the xenograft and mouse models suitable for investigating new therapeutic compounds for the treatment of B-cell malignancies. Curr Pharm Des. (2008) 14:2023–39. 10.2174/138161208785294591 18691113

[16] Zullo K Amengual JE O'Connor OA Scotto L. Murine models in mantle cell lymphoma. Best Pract Res Clin Haematol. (2012) 25:153–63. 10.1016/j.beha.2012.04.009 22687451

[17] Zandvliet M. Canine lymphoma: a review. Vet Q. (2016) 36:76–104. 10.1080/01652176.2016.1152633 26953614

[18] LeBlanc AK Mazcko CN Khanna C. Defining the value of a comparative approach to cancer drug development. Clin Cancer Res. (2016) 22:2133–8. 10.1158/1078-0432.CCR-15-2347 26712689 PMC5111620

[19] Saba C Paoloni M Mazcko C Kisseberth W Burton JH Smith A. A comparative oncology study of iniparib defines its pharmacokinetic profile and biological activity in a naturally-occurring canine cancer model. PLoS ONE. (2016) 11:e0149194. 10.1371/journal.pone.0149194 26866698 PMC4751284

[20] Kletting P Maass C Reske S Beer AJ Glatting G. Physiologically based pharmacokinetic modeling is essential in 90Y-labeled anti-CD66 radioimmunotherapy. PLoS ONE. (2015) 10:e0127934. 10.1371/journal.pone.0127934 26010360 PMC4444288

[21] Maass C Kletting P Bunjes D Mahren B Beer AJ Glatting G. Population-based modeling improves treatment planning before (90)Y-labeled anti-CD66 antibody radioimmunotherapy. Cancer Biother Radiopharm. (2015) 30:285–90. 10.1089/cbr.2015.1878 26172337

[22] Rutgen BC Hammer SE Gerner W Christian M de Arespacochaga AG Willmann M. Establishment and characterization of a novel canine B-cell line derived from a spontaneously occurring diffuse large cell lymphoma. Leuk Res. (2010) 34:932–8. 10.1016/j.leukres.2010.01.021 20153049

[23] Diab M Nguyen F Berthaud M Maurel C Gaschet J Verger E. Production and characterization of monoclonal antibodies specific for canine CD138 (syndecan-1) for nuclear medicine preclinical trials on spontaneous tumours. Vet Comp Oncol. (2017) 15:932–51. 10.1111/vco.12233 27076401

[24] Matrisian LM Bowden GT Krieg P Furstenberger G Briand JP Leroy P. The mRNA coding for the secreted protease transin is expressed more abundantly in malignant than in benign tumors. Proc Natl Acad Sci USA. (1986) 83:9413–7. 10.1073/pnas.83.24.9413 3540938 PMC387148

[25] Fraker PJ Speck JC Jr. Protein and cell membrane iodinations with a sparingly soluble chloroamide, 1,3,4,6-tetrachloro-3a,6a-diphrenylglycoluril. Biochem Biophys Res Commun. (1978) 80:849–57. 10.1016/0006-291X(78)91322-0 637870

[26] Nikula TK Curcio MJ Brechbiel MW Gansow OA Finn RD Scheinberg DA. A rapid, single vessel method for preparation of clinical grade ligand conjugated monoclonal antibodies. Nucl Med Biol. (1995) 22:387–90. 10.1016/0969-8051(94)00126-5 7627155

[27] Tembhare PR Marti G Wiestner A Degheidy H Farooqui M Kreitman RJ. Quantification of expression of antigens targeted by antibody-based therapy in chronic lymphocytic leukemia. Am J Clin Pathol. (2013) 140:813–8. 10.1309/AJCPYFQ4XMGJD6TI 24225748 PMC4698892

[28] Mattes MJ Shih LB Govindan SV Sharkey RM Ong GL Xuan H. The advantage of residualizing radiolabels for targeting B-cell lymphomas with a radiolabeled anti-CD22 monoclonal antibody. Int J Cancer. (1997) 71:429–35. 10.1002/(sici)1097-0215(19970502)71:3<429::aid-ijc21>3.0.co;2-9 9139880

[29] John B Herrin BR Raman C Wang YN Bobbitt KR Brody BA. The B cell coreceptor CD22 associates with AP50, a clathrin-coated pit adapter protein, via tyrosine-dependent interaction. J Immunol. (2003) 170:3534–43. 10.4049/jimmunol.170.7.3534 12646615

[30] Geissler F Anderson SK Venkatesan P Press O. Intracellular catabolism of radiolabeled anti-mu antibodies by malignant B-cells. Cancer Res. (1992) 52:2907–15. 1581908

[31] Naruki Y Carrasquillo JA Reynolds JC Maloney PJ Frincke JM Neumann RD. Differential cellular catabolism of 111In, 90Y and 125I radiolabeled T101 anti-CD5 monoclonal antibody. Int J Rad Appl Instrum B. (1990) 17:201–7. 10.1016/0883-2897(90)90148-T 1692819

[32] O'Reilly MK Tian H Paulson JC. CD22 is a recycling receptor that can shuttle cargo between the cell surface and endosomal compartments of B cells. J Immunol. (2011) 186:1554–63. 10.4049/jimmunol.1003005 21178016 PMC3024466

[33] Shan D Press OW. Constitutive endocytosis and degradation of CD22 by human B cells. J Immunol. (1995) 154:4466–75. 7722303

[34] Lesley J Schulte R Woods J. Modulation of transferrin receptor expression and function by anti-transferrin receptor antibodies and antibody fragments. Exp Cell Res. (1989) 182:215–33. 10.1016/0014-4827(89)90293-0 2653853

[35] Rylova SN Stoykow C Del Pozzo L Abiraj K Tamma ML Kiefer Y. The somatostatin receptor 2 antagonist 64Cu-NODAGA-JR11 outperforms 64Cu-DOTA-TATE in a mouse xenograft model. PLoS ONE. (2018) 13:e0195802. 10.1371/journal.pone.0195802 29668724 PMC5906006

[36] Sharkey RM Behr TM Mattes MJ Stein R Griffiths GL Shih LB. Advantage of residualizing radiolabels for an internalizing antibody against the B-cell lymphoma antigen, CD22. Cancer Immunol Immunother. (1997) 44:179–88. 10.1007/s002620050371 9191878 PMC11037676

[37] Bergeron LM McCandless EE Dunham S Dunkle B Zhu Y Shelly J. Comparative functional characterization of canine IgG subclasses. Vet Immunol Immunopathol. (2014) 157:31–41. 10.1016/j.vetimm.2013.10.018 24268690

